# Epidemiologic and Clinical Characteristics of Intentional Injuries among Cases Admitted to Sina Hospital: Affiliated with the National Trauma Registry of Iran

**DOI:** 10.34172/jrhs.2023.122

**Published:** 2023-09-29

**Authors:** Mahgol Sadat Hassan Zadeh Tabatabaei, Vali Baigi, Mohammadreza Zafarghandi, Vafa Rahimi-Movaghar, Sobhan Pourmasjedi, Armin Khavandegar, Khatereh Naghdi, Payman Salamati

**Affiliations:** ^1^Sina Trauma and Surgery Research Center, Sina Hospital, Tehran University of Medical Sciences, Tehran, Iran

**Keywords:** Registries, Self-injurious behavior, Suicide, Violence, Iran

## Abstract

**Background:** Intentional injuries, including self-harm, suicide, conflict, and interpersonal violence are a significant public health concern in Iran, but they have not been adequately documented. This study aimed to investigate intentional injuries in cases admitted to Sina Hospital in Tehran, Iran, affiliated with the National Trauma Registry of Iran.

**Study Design:** A retrospective cohort study.

**Methods:** A registry-based study on the characteristics of 852 intentional injury cases was conducted from 2016 to 2023. Information on various aspects, including baseline characteristics, injury characteristics, and injury outcomes was compared between groups of self-harm/suicide, conflict/interpersonal violence, and others (abuse and legal prosecution).

**Results:** Of 6,692 registered trauma cases, 852 (12.7%) had intentional injuries. Men accounted for 92 (77.3%) self-harm/suicide and 650 (96.4%) conflict/interpersonal violence cases (*P*<0.001). Self-harm/ suicide mostly occurred at home in 89 (74.8%) cases, while 73 (10.8%) conflict/interpersonal violence cases happened at home (*P*<0.001). Falls were the cause of trauma in 12 (10.1%) self-harm/suicide cases compared to 7 (1.0%) conflict/interpersonal violence cases (*P*<0.001). Furthermore, blunt trauma was the cause of trauma in one (0.8%) case of self-harm/suicide and 66 (9.8%) conflict/interpersonal violence cases (*P*<0.001). Moreover, 14 (11.8%) self-harm/suicide and 34 (5.0%) conflict/interpersonal violence cases required ventilation (*P*=0.010). Additionally, 74 (8.7%) intentional injury cases had multiple traumas, which were seen in nine (7.6%) self-harm/suicide and 58 (8.6%) conflict/interpersonal violence cases (*P*<0.001).

**Conclusion:** Men were the majority of self-harm/suicide and conflict/interpersonal violence cases. Self-harm/suicide incidents mostly occurred at home and resulted in more injuries from falls, while conflict/ interpersonal violence resulted in increased blunt traumas and multiple traumas.

## Background

 Damage to the human body by unintentional or deliberate exposure to an energy source is an injury.^1^ Each year, 4.4 million individuals worldwide die from injuries, accounting for nearly 8% of all fatalities.^2^ Injuries dramatically increase morbidity, and both together are estimated to account for approximately 10% of years lived with disability (YLDs).^3^ We should consider injuries as a worldwide health concern having a tremendous negative health and economic impact on nations.^2,4,5^ High-risk behaviors committed to harm self or “others” are considered intentional injuries.^6,7^ Homicide, self-harm, suicide, conflicts, interpersonal violence, and legal prosecution are examples.^4,8^ Intentional injuries claim the lives of 1.25 million people annually, accounting for 28% of the total 4.4 million injury-related deaths.^2^ Injury morbidity can leave the patients permanently or temporarily incapacitated, affecting a far larger population.^2,9^

 There is an uneven distribution of intentional injuries within or between nations which are influenced by various factors. Developing nations are places in which the majority of intentional injury statistics are reported. According to previous research, this issue is escalating quickly in developing countries although most of them lack adequate data on the morbidity and mortality of intentional injuries.^10,11^ Alcohol abuse, drug addiction, and firearm access have all been identified as risk factors for intentional injuries.^6^ Poor living and working conditions, a lack of access to trauma-informed care, unemployment, and drug and alcohol abuse are the key contributing factors in developing nations.^12^

 The trauma registry is an important analysis tool that captures trauma care’s epidemiology, management, and outcomes. It enables governments to access important data that can be used in trauma management improvement.^13^ The trauma registry’s data can be used as a reliable source of clinical and epidemiological information on trauma cases. To our knowledge, there are a few studies specifically focused on intentional injuries in the Iranian population. Furthermore, there is insufficient data on the scale, characteristics, and morbidity and mortality rates of intentional injuries in Iran.^14^ Hence, this study was conducted to analyze the data from the cases admitted to Sina Hospital, Tehran, Iran, affiliated with the National Trauma Registry of Iran (NTRI), to assess the clinical characteristics of intentional injuries among the registered cases.

## Methods

###  Study design

 We carried out a registry-based investigation among cases with intentional injuries between September 17, 2016 and January 21, 2023. The National Trauma Research Institute (NTRI) is a multicenter registry run by the cooperation of some of the country’s main trauma centers. The Sina Trauma and Surgery Research Center initially launched the registry at Sina Hospital, Tehran, Iran, in 2015.^15^ Sina hospital is one of the collaborating centers (the main center) of the NTRI. The NTRI’s registration process, a minimal dataset, and data quality assurance have all been covered in previous articles. We included all intentional injury cases according to the International Classification of Diseases, Tenth Revision (ICD-10) who were referred to Sina Hospital and matched one of the NTRI inclusion criteria. These criteria included hospitalization for more than 24 hours, dying from an injury on the first day of admission, or transferring from other hospitals’ intensive care units (ICUs).^15^ The ICD-10 codes used were X60-Y09, Y87.0, and Y87.1, X60-X84 for intentional self-harm, X85-Y09 for assault, Y87.0 for sequelae of intentional self-harm, and Y87.1 for sequelae of assault.

###  Data collection

 The registration process was divided into various phases. In the first step, the hospital information system (HIS) identified patients who met the criteria. In the next step, three qualified and trained nurses retrieved patients’ data from HIS and in-person interviews with the patients. The data would be extracted from their companions or hospital files if a patient could not be interviewed. To ensure accurate and consistent data collection, the interviewers were trained for three days on the registry process and platform. The questionnaire contained 99 various variables, which were divided into eight different sections. The questionnaire form was discussed in detail in earlier articles.^16^ Then, the gathered data were entered into the NTRI database portal by the three experienced registrars with medical backgrounds. Finally, a professional physician reviewed and verified the correctness and completeness of the data. The data collection process was also discussed in detail in the NTRI pilot phase of the previous article.^15^

 We took into account a number of significant aspects of data quality to make sure the data acquired for this study were of high quality. Trained registrars provided all relevant information and sought to address any concerns with attending doctors, patients, or their relatives to achieve completeness. Verification and final registration were postponed until all fields were filled. To assure accuracy, we gathered injury characteristics data directly from credible sources such as interviews and patient files. We employed logic programming in the NTRI software to assure correctness and consistency, including context rules, syntactic rules, temporal rules, and rules for permissible value range. The supervisor was in charge of all duties and tasks and randomly examined the forms to ensure proper completion and review. We made sure that the technical elements such as data structures, data layouts, and software development were the same to ensure data compatibility.^15^ A previous study conducted in Sina Hospital found that the data quality and patient coverage are high. Additionally, the reliability of most variables was acceptable.^17^

###  Variables

 We categorized intentional injury into three groups: conflict/interpersonal violence, self-harm/suicide, and the others (i.e., abuse and legal prosecution). Self-harm was defined as purposeful self-poisoning or self-injury.^18^ We analyzed variables, including age, gender, marital status, education, illicit drug, alcohol, and sedative consumption, place of occurrence, cause of trauma, body region, injury severity score (ISS), Glasgow Coma Scale (GCS), ICU admission, length of stay (LOS), mechanical ventilation, and death. No formal education (illiterate), primary education, lower secondary, upper secondary, and tertiary education (university education) constituted the five groups used to classify the cases’ education. The term “sedative drug” encompassed both prescription and over-the-counter medications, including benzodiazepines, barbiturates, and antihistamines, among others. The place of injury occurrence was divided into two groups: inside and outside the home. Causes of trauma were stab/cut, blunt injuries, poisoning, firearms, road traffic crashes, falls, and miscellaneous (i.e., animal bites, traumatic asphyxia, electrical injury, and blast injury). The severity of an injury to any part of the body was assessed using an abbreviated injury scale (AIS). For assessing the severity of damage to any area of the body, AIS assigns a number from 1 to 6 (minor AIS = 1, moderate AIS = 2, serious AIS = 3, severe AIS = 4, critical AIS = 5, and maximal trauma AIS = 6). The ISS was used to quantify the severity of injuries in particular areas. The three most severely injured body regions’ highest AIS scores were squared and added together to determine the ISS. This study defined mild injury as an ISS of 1 to 8 and moderate/severe injury as an ISS of 9 or higher.^19-21^ The GCS score was obtained by combining the scores for the eye-opening response, verbal response, and motor response. It ranged from 3 (deep coma) to 15 (fully conscious and alert). Furthermore, serious head damage was defined as a GCS score of 8 or below, moderate head injury as a GCS score of 9 to 12, and mild head injury as a GCS score of 13 to 15.^22^ We defined multiple traumas as having two or more trauma injuries with AIS ≥ 3.^23^

###  Statistical analysis 

 Quantitative variables with normal distribution were described using the mean and standard deviation. Frequency and percentage were used to describe nominal and categorical variables, and ANOVA was utilized to compare quantitative variables among different intentional injuries. In addition, chi-square was used to assess the association between nominal and categorical variables with types of intentional injury. Pairwise comparisons were then conducted using Bonferroni’s test, and *P*< 0.05 was deemed statistically significant. Statistical analyses were conducted using the STATA software version 15.0 (Stata Corp, College Station, TX, USA).

## Results

**Figure 1:**
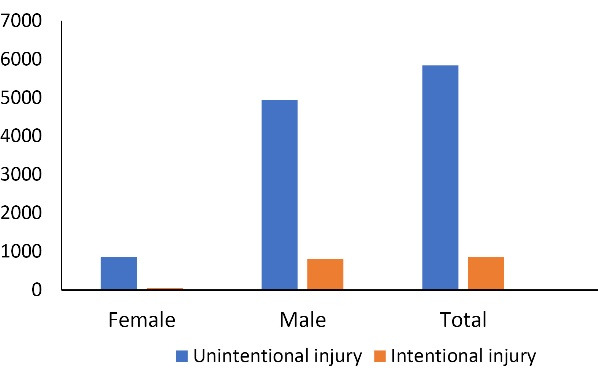
Distribution of trauma cases by intent in the patients admitted at Sina hospital, Tehran, Iran. The figure depicts the distribution of intentional and unintentional injuries in the patients admitted to Sina hospital from September 17, 2016 to January 21, 2023. The x-axis illustrates the gender categories of the trauma cases with 5747 males and 945 females. The y-axis illustrates the number of intentional and unintentional injuries out of the total and for each gender. Out of 6692 trauma cases, 852 (12.7%) had intentional injuries. In addition, 799 (13.9%) males and 53 (5.6%) females had intentional injuries

 There were 852 (12.7%) intentional injuries among 6,692 cases. Men accounted for 799 (93.8%) cases of intentional injuries. Figure 1 features the distribution of intentional and unintentional injuries across our study population based on gender.

 Cases’ ages ranged from 13 to 89 years, with a mean ( ± SD) of 31.3 ( ± 11.6) years. Cases between 18 and 49 years committed 739 (86.7%) cases of intentional injuries. The three categories of intentional injuries had significantly different age distributions (*P* = 0.001).

 Significant gender differences were found between the categories of intentional injuries (*P* < 0.001). The majority of those who committed self-harm/suicide were men. Moreover, men comprised 96.4% of those involved in conflict/interpersonal violence. Further data on baseline characteristics of different groups of intentional injuries are outlined in Table 1.

**Table 1:** Baseline characteristics of intentional injuries in patients under study

**Variables**	**Self-harm/suicide** **(n=119)**	**Conflict/interpersonal** **violence (n=674)**	**Others** **(n=59)**	**Total** **(n=852)**	***P *****value**
Age group (y)					0.010
< 18	6	44	5	55	
18-49	94^a^	595^b^	50^a,b^	739	
≥ 50	19^a^	35^b^	4^a,b^	58	
Gender					0.001
Female	27^a^	24^b^	2^a^	53	
Male	92^a^	650^b^	57^b^	799	
Education					0.071
No formal	8	35	1	44	
Primary	9	61	12	82	
Lower secondary	28	169	10	207	
Upper secondary	46	302	27	375	
Tertiary	18	72	5	95	
Marital status					0.160
Single	67	402	39	508	
Divorced/widow	45	252	18	315	
Married	7	15	1	23	
Alcohol consumption					0.488
Yes	6	19	3	28	
No	108	638	54	800	
Missing	5	17	2	24	
Drug consumption					0.492
Yes	6	42	6	54	
No	107	613	51	771	
Missing	6	19	2	27	
Sedative consumption					0.077
Yes	4	7	2	13	
No	109	652	55	816	
Missing	6	15	2	23	

*Note.* Others: Abuse and legal prosecution; a and a denotes a subset of intentional injuries categories whose column proportions do not differ significantly from each other at the 0.05 level.

 The most common cause of trauma in self-harm/suicides was stab/cut (81.5%), followed by falls (10.1%) and firearms (2.5%). Stab/cut (84.3%), blunt injuries (9.8%), and firearms (3.4%) were the most common trauma mechanisms used in conflict/interpersonal violence. However, no cases of poisoning were found in the conflict/interpersonal violence and “others” groups. The frequency of blunt injuries varied significantly between the three types of intentional injuries and was most common among those in conflict/interpersonal violence (*P* < 0.001). Falls were significantly more observed in cases who committed self-harm/suicide (*P* < 0.001). Furthermore, there were no significant differences in severity between conflict/interpersonal violence and self-harm/suicide categories (Table 2).

**Table 2:** The relationship between the injury characteristics and the type of intentional injuries

**Variables**	**Self-harm/ suicide** **(n=119)**	**Conflict/interpersonal** **violence (n=674)**	**Others** **(n=59)**	**Total** **(n=85)**	***P *****value**
Place of intentional injury					0.001
Inside home	89^a^	73^b^	2^b^	164	
Outside home	30	601	57	688	
Cause of trauma					0.001
Blunt	1^a^	66^b^	2^a,b^	69	
Road traffic crashes	2^a^	9^a^	5^b^	16	
Poisoning	3^a^	0^b^	0^a,b^	3	
Fall	12^a^	7^b^	4^a^	23	
Firearms	3^a^	23^a^	17^b^	43	
Stab/cut	97^a^	568^a^	31^b^	696	
Miscellaneous	1	1	0	2	
Injury severity score					0.373
Mild (1-8)	112	614	52	778	
Moderate/severe ( ≥ 9)	7	60	7	74	

*Note.* Others: Abuse and legal prosecution; * Each superscript letter (a, b, and a,b) denotes a subset of intentional injuries categories whose column proportions do not differ significantly from each other at the 0.05 level.

Table 3 illustrates the relationship between body regions and the type of intentional injuries. The most common injuries were to the upper extremity (451 cases), lower extremity (92 cases), and thorax (89 cases). Moreover, 74 cases had multiple traumas. The most common injuries in self-harm/suicide cases occurred in their upper extremity (64.7%), abdomen (10.1%), and head/face/neck (9.2%) regions. The most often damaged regions during conflict/interpersonal violence were the upper extremities (52.1%), thorax (12.3%), and head/face/neck (10.8%). Of the cases who experienced conflict/interpersonal violence, 12.3% suffered thorax injuries, while only 1.7% of those who self-harmed or committed suicide suffered thorax injuries (*P* < 0.001). Furthermore, self-harm/suicide cases had a higher percentage (64.7%) of upper extremity injuries compared to conflict/interpersonal violence cases (52.1%) (*P* < 0.001).

**Table 3:** The relationship between the body regions injured and the type of intentional injuries

**Variables**	**Self-harm/ suicide** **n=119**	**Conflict/interpersonal** **violence n=674**	**Others** **n=59**	**Total** **n=852**
Head/neck/face	11	73	4	88
Thorax	2^a^	83^b^	4^a,b^	89
Abdomen	12	39	2	53
Spine	0	5	0	5
Upper extremity	77^a^	351^b^	23^b^	451
Lower extremity	8^a^	65^a^	19^b^	92
Multiple trauma	9	58	7	74

*Note.* Others: Abuse and legal prosecution; *P*-vale = 0.001; a and b denote a subset of intentional injuries categories whose column proportions do not differ significantly from each other at the 0.05 level.

Table 4 depicts the outcomes of intentional injuries. The mean ± SD LOS for intentional injuries was 122.0 ± 148.0 hours. Self-harm/suicide and conflict/interpersonal violence cases had a mean ± SD LOS of 112.7 ± 95.0 and 122.2 ± 157.8 hours, respectively (*P* = 0.68). Moreover, 16 documented deaths were reported in four cases who committed self-harm/suicide, and 11 cases died due to conflict/interpersonal violence and one legal prosecution. While 11.8 % of cases who were admitted to the hospital as a result of self-harm/suicides were ventilated, only 5% of those who were admitted due to conflict/interpersonal violence needed ventilators (*P* = 0.01). Furthermore, no significant differences were observed in ICU admission, mortality, and LOS between the three groups of intentional injuries.

**Table 4:** The relationship between injury outcome and the type of intentional injuries

**Variables**	**Self-harm/ suicide (n=119)**	**Conflict/interpersonal** **violence (n=674)**	**Others** **(n=59)**	**Total** **(n=852)**	***P *****value**
Mechanical ventilation (Yes)	14^a^	34^b^	2 ^a,b^	50	0.011
ICU admission	19	73	5	97	0.204
Death	4	11	1	16	0.438

*Note.* Others: Abuse and legal prosecution;ICU: Intensive care Unit; LOS: Length of stay; a and b denote a subset of intentional injuries categories whose column proportions do not differ significantly from each other at the 0.05 level.

## Discussion

 While intentional injuries are a significant public health concern in Iran, there is currently a lack of comprehensive data on the characteristics and outcomes of intentional injury cases. This study aimed to fill this knowledge gap by investigating intentional injuries in cases admitted to Sina Hospital in Tehran, Iran, using data from the NTRI.

 The results of the present study showed that intentional injuries accounted for 12% of the overall 6,692 cases admitted at Sina Hospital over nearly six years, with men being the most affected group. In this study, men were responsible for most intentional injuries. These findings are consistent with those reported elsewhere.^24^ In Yin and colleagues’ study, male gender was identified as a risk factor for intentional injury.^25^ Shirah et al carried out a prospective cohort study of the clinical trends and reported therapeutic results of 252 individuals with intentional injuries with a mean ± SD age of 34.2 ± 9.4 years, more than half of whom were males.^26^ In this study, men comprised the vast majority of self-harm/suicide patients. In the low- and middle-income countries (LMICs), the male-to-female suicide ratio was 1.5 men for every woman.^27^ Women committed suicides and attempted suicides at higher rates than men, according to research conducted by Rahimi-Movaghar et al from 2005 to 2008 in Iran.^28^ According to Rezaeian’s study, Females from Eastern Mediterranean countries were more likely to commit suicide than males.^29^ According to the research conducted by Niyaraq Nobakht et al on self-harm in Iranian adults, male individuals were engaged more frequently in self-harm than female individuals.^30^

 Cases aged between 18 and 49 made up the majority of our intentional injury cases. This finding aligns with the literature indicating that young individuals are particularly vulnerable to intentional injuries. Gal et al implemented a study to assess the epidemiology of assault and self-harm injuries in a large Romanian Emergency Department. It delineated that most patients were between 15 and 44.^31^ These findings imply that initiatives for education, preparing health care providers to deal with intentional injuries, and preventative efforts should concentrate on young adult men.

 Alcohol consumption was found in 5% of self-harm/suicide cases and 2.8% of conflict/interpersonal violence cases in the present study. Gal et al outlined that alcohol use was observed in 16.3% of self-harm patients and 21.4% of assault victims.^31^ Additionally, the consumption of illicit drugs, alcohol, and sedatives before the injury did not significantly differ between the intentional injury groups in our study. This finding differs from most previous studies. Several studies have shown a link between aggression and alcohol intake.^32,33^ Some hypotheses are proposed, and some of these are impulsivity, cognitive impairment, and attention impacts.^34^ According to the World Health Organization (WHO) worldwide status report on alcohol in 2004, the estimated total alcohol consumption per resident aged 15 and older in liters of absolute alcohol was 1.3 liters in the Eastern Mediterranean countries such as Iran and Saudi Arabia.^33^ Shirah et al reported that although alcohol consumption is forbidden in Saudi Arabian society, it is reported in 12.3% of patients with intentional injuries.^26^ We think that this disparity can be attributable to our Islamic country’s religious peculiarities. In Iran, the use of alcoholic beverages and illegal narcotics is outlawed. Due to this condition, only a few individuals in our study reported the consumption of alcohol and other illicit drugs prior to the injury. It is possible that selective underreporting has occurred.

 The majority of our intentional injury cases happened outside the home. Previous studies delineated similar findings.^26,35,36^ Moreover, most of the self-harm/suicide cases of the current study happened at home. Likewise, in the study by Gal et al, more than 88% of self-harm injuries occurred at home or in a residential facility, similar to our study.^31^

 We had 119 self-harm/suicide cases, 674 interpersonal violence/conflict cases, and 59 cases classified as the “others” group. There were 32 cases of abuse and 27 with legal prosecution in the “others” group. We did not have access to the type of abuse in the present study. We noticed 16 cases of road traffic crashes with intentional injuries, among which two cases were in the self-harm/suicide group. One person threw himself in front of the oncoming subway, and one person was a courier who tried to commit suicide while riding the motorcycle. There were nine cases of conflict/interpersonal violence, and pedestrians were intentionally hit by a car or motorcycle. Five cases in the group of the “others” were injured in the police chase. The most common cause of trauma in our self-harm/suicides was stab/cut, followed by falls. Stab/cut and blunt injuries were the most common trauma mechanisms in our study’s conflict/interpersonal violence cases. These findings are in line with the previous studies. Yin and colleagues’ study comprised 85 677 Chinese children and adolescents. It indicated that the most prevalent types of injury in violent attacks were cuts and blunt injuries. Cuts were the most common trauma mechanism in patients injured by self-mutilation and suicides in the current study.^25^ Swarnkar et al also demonstrated a substantial difference in injury mechanisms between self-harm and assault patients in India. The most common injury mechanism among self-harm patients was cutting and piercing.^35^ In Shirah and colleagues’ study, sharp objects were also the most often utilized weapons in intentional injuries.^26^

 Falls were significantly more observed in cases who committed self-harm/suicide (*P* < 0.001). The result is consistent with other studies. Gal et al showed significant differences in the mechanisms of injury between assault and self-harm injuries. Additionally, they presented that suffocation and falls were frequent causes of self-harm but not for assault injuries.^31^ This study contained only a few firearms cases in self-harm/suicide and conflict/interpersonal violence cases. The reason may be the Iranian government’s tight prohibitions on personal firearms, which prevent them from being widely available.

 The most common injuries in self-harm/suicide cases were to their upper extremities and abdomen. Izawa et al found that the abdomen and extremities were the most prevalent injury sites among self-inflicted penetrating injuries. Interestingly, there were more abdomen injuries than extremities in this study which were attributed to a different means of attempting suicide in Japanese society than in ours.^37^ In the study by Swarnkar et al, the upper extremities were the most commonly injured areas in self-harm.^35^

 We have used the ICD-10 codes for intentional injuries in this study, which provide a less detailed and updated classification system compared to the ICD-11 codes. The ICD-11 includes new categories for intentional injuries such as intentional self-harm by being stepped on or crushed by an animal, which were not available in the ICD-10. Additionally, the ICD-11 codes provide more detailed information on the severity and type of injuries as well as the intent behind the injury (e.g., self-harm vs. assault).

 Suicide and self-harm are extremely sensitive and essential topics that affect everyone, and that is why studies dedicated to them are scarce. Suicide is frequently misclassified as an accident or another cause of death in death certificates. Suicide and suicide attempt case registration is a complicated and detailed process that is not always adequately documented, and LMICs face the majority of the worldwide suicide burden.^27^ Suicide prevention programs should be implemented to address the noticeable rates of self-harm/suicide observed in LMICs. Through the years, violence has been seen as a serious public health problem that has to be addressed by public health experts.^6^ All in all, this study investigated self-harm/suicide and conflict/interpersonal violence cases in one of Iran’s main trauma centers, which is an important topic requiring attention and prevention efforts.

 It is important to identify some of the study’s limitations and strengths. This study is the first comprehensive analysis of intentional injuries based on the NTRI’s data. Moreover, we did not have access to information on all types of intentional injuries. For instance, there were no categories for violence against children, violence against women, violence against the elderly, and sexual violence in this study. Since suicide and suicide attempts are sensitive issues, there may have been underreported incidents. The fact that the perpetrators of the incident or other participants in cases of interpersonal violence or conflict may not be taken to the hospital was another restriction that could impact the outcomes. Another limitation of this study is related to the nature of data collection, which involves measuring the consumption of alcohol, drugs, and sedatives. Due to the specific characteristics of these variables, there were limited conditions under which bias analysis or sensitivity analysis could be effectively conducted.

**Highlights**

- Men were the majority of self-harm/suicide and conflict/interpersonal violence cases.
- Self-harm/suicide mostly occurred at home, resulting in falls, while conflict/interpersonal violence resulted in blunt trauma and multiple traumas.
- Upper extremities were more common injured body regions in self-harm/suicide cases, while conflict/interpersonal violence cases had more injuries in thorax.
- Self-harm/suicide cases needed more ventilation than conflict/interpersonal violence cases.

## Conclusion

 This study provided a registry-based investigation of the patterns of intentional injuries among cases admitted to Sina Hospital. The cause of trauma, body region, and mechanical ventilation differed markedly between the self-harm/suicide and conflict/interpersonal violence groups. More cases who self-harmed or committed suicide had trauma related to falls compared to conflict/interpersonal violence victims. Cases who attempted self-harm/suicide needed more frequent ventilation than cases with conflict/interpersonal violence.

## Acknowledgements

 We are grateful to all colleagues who participated in this study.

## Authors’ Contribution

**Conceptualization:** Payman Salamati, Mahgol Sadat Hassan Zadeh Tabatabaei.

**Data curation:** Sobhan Pourmasjedi, Khatereh Naghdi.

**Formal analysis:** Vali Baigi.

**Funding acquisition:** Mohammadreza Zafarghandi.

**Investigation:** Armin Khavandegar, Sobhan Pourmasjedi.

**Methodology:** Vali Baigi.

**Project administration:** Payman Salamati, Mohammadreza Zafarghandi.

**Resources: **Mohammadreza Zafarghandi.

**Software:** Khatereh Naghdi.

**Supervision:** Vafa Rahimi-Movaghar, Payman Salamati.

**Validation:** Vafa Rahimi-Movaghar, Sobhan Pourmasjedi.

**Visualization:** Mahgol Sadat Hassan Zadeh Tabatabaei, Vali Baigi.

**Writing–original draft:** Mahgol Sadat Hassan Zadeh Tabatabaei.

**Writing–review & editing:** Armin Khavandegar.

## Competing Interests

 The authors declare no potential competing interests.

## Ethical Approval

 This study was approved by the ethics committee of Sina Hospital, Tehran University of Medical Sciences (Approval ID: IR.TUMS.SINAHOSPITAL.REC.1399.090). All methods were performed in accordance with the ethical standards as laid down in the Declaration of Helsinki and its later amendments or comparable ethical standards. Written informed consent was also obtained from all participants or a parent and/or legal guardian if participants were under 16.

## Funding

 Sina Trauma and Surgery Research Center financially supported the study under the plan of the National Trauma Registry of Iran.
